# Japanese subgroup analysis of a phase III study of S-1 versus docetaxel in non-small cell lung cancer patients after platinum-based treatment: EAST-LC

**DOI:** 10.1007/s10147-019-01396-z

**Published:** 2019-03-04

**Authors:** Shunichi Sugawara, Kazuhiko Nakagawa, Nobuyuki Yamamoto, Hiroshi Nokihara, Yuichiro Ohe, Makoto Nishio, Toshiaki Takahashi, Koichi Goto, Makoto Maemondo, Yukito Ichinose, Takashi Seto, Hiroshi Sakai, Akihiko Gemma, Fumio Imamura, Masato Shingyoji, Hideo Saka, Akira Inoue, Koji Takeda, Isamu Okamoto, Katsuyuki Kiura, Satoshi Morita, Tomohide Tamura

**Affiliations:** 1 Department of Pulmonary Medicine, Sendai Kousei Hospital, Miyagi, Japan; 2 Department of Medical Oncology, Faculty of Medicine, Kindai University, Osaka, Japan; 3 Third Department of Internal Medicine, Wakayama Medical University, Wakayama, Japan; 4 Department of Thoracic Oncology, National Cancer Center Hospital, Tokyo, Japan; 5 Department of Thoracic Medical Oncology, The Cancer Institute Hospital of Japanese Foundation for Cancer Research, Tokyo, Japan; 6 Division of Thoracic Oncology, Shizuoka Cancer Center, Shizuoka, Japan; 7 Department of Thoracic Oncology, National Cancer Center Hospital East, Kashiwa, Japan; 8 Department of Respiratory Medicine, Miyagi Cancer Center, Miyagi, Japan; 9 Department of Cancer Information Research, National Kyushu Cancer Center, Clinical Research Institute, Fukuoka, Japan; 10 Department of Thoracic Oncology, National Kyushu Cancer Center, Fukuoka, Japan; 11 Department of Thoracic Oncology, Saitama Cancer Center, Saitama, Japan; 12 Department of Pulmonary Medicine and Oncology, Graduate School of Medicine, Nippon Medical School, Tokyo, Japan; 13 Department of Thoracic Oncology, Osaka International Cancer Institute, Osaka, Japan; 14 Division of Respirology, Chiba Cancer Center, Chiba, Japan; 15 Department of Respiratory Medicine and Medical Oncology, National Hospital Organization Nagoya Medical Center, Aichi, Japan; 16 Department of Palliative Medicine, Tohoku University School of Medicine, Miyagi, Japan; 17 Department of Medical Oncology, Osaka City General Hospital, Osaka, Japan; 18 Research Institute for Diseases of the Chest, Graduate School of Medical Sciences, Kyushu University, Fukuoka, Japan; 19 Department of Hematology, Oncology, and Respiratory Medicine, Okayama University Graduate School of Medicine, Okayama, Japan; 20 Department of Biomedical Statistics and Bioinformatics, Kyoto University Graduate School of Medicine, Kyoto, Japan; 21 Thoracic Center, St Luke’s International Hospital, 9-1 Akashi-cho, Chuo-ku, Tokyo, 104-8560 Japan

**Keywords:** Tegafur–gimeracil–oteracil, Second line, Third line, Chemotherapy, Japan, Phase III

## Abstract

**Introduction:**

The East Asia S-1 Trial in Lung Cancer (EAST-LC) was a randomized phase III study conducted in East Asia that demonstrated the non-inferiority of S-1 to docetaxel in previously treated patients with advanced non-small cell lung cancer (NSCLC). Here, we reported the results of the Japanese subgroup treated with docetaxel 60 mg/m^2^, the standard dosage in Japan.

**Patients and methods:**

Patients were randomized 1:1 to receive either S-1 or docetaxel. The primary endpoint was overall survival (OS); the secondary endpoints included progression-free survival (PFS), response rate (RR), quality of life (QOL), and safety.

**Results:**

Patient characteristics in the Japanese subgroup (*n* = 724) were similar to those in the overall EAST-LC population. Median OS was 13.4 months in the S-1 group and 12.6 months in the docetaxel group. In pemetrexed-pretreated patients, OS with S-1 was similar to that with docetaxel. Median PFS was 2.9 and 3.0 months in the S-1 and docetaxel groups, respectively. RR was 9.4% and 10.3% in the S-1 and docetaxel groups, respectively. The QOL of patients treated with S-1 was better compared with that of patients treated with docetaxel. Decreased appetite and diarrhea were more common in the S-1 group, whereas the frequency of neutropenia and febrile neutropenia was markedly higher in the docetaxel group.

**Conclusions:**

This Japanese subgroup analysis showed that S-1 had similar efficacy to docetaxel in patients with previously treated advanced NSCLC. These results are similar to those of the overall EAST-LC population.

**Electronic supplementary material:**

The online version of this article (10.1007/s10147-019-01396-z) contains supplementary material, which is available to authorized users.

## Introduction

Lung cancer is the most prevalent cancer worldwide, and it was responsible for 1.69 million deaths in 2015 [1]. The most common form of lung cancer is non-small cell lung cancer (NSCLC), which accounts for approximately 85% of all cases [2]. At the time of initial diagnosis, NSCLC is locally advanced or distant metastases are present in 40–50% of patients [3]. Patients with advanced or metastatic NSCLC have a poor prognosis compared with patients with other cancers [4].

In major treatment guidelines [5, 6], including Japanese recommendations [7], platinum-based double-agent chemotherapy is the mainstay of first-line therapy for stage IV NSCLC in patients without any specifically identified oncogenic driver [e.g., endothelial growth factor receptor (EGFR) or anaplastic lymphoma kinase fusion] or for whom immune checkpoint inhibitors are inappropriate. Furthermore, current guidelines recommend non-platinum-based chemotherapy with docetaxel (with or without ramucirumab) or pemetrexed in the second-line setting [5–7]. However, there is an unmet need for effective treatment options in previously treated patients for whom targeted therapies or immunotherapies are inappropriate or ineffective. An analysis of systemic treatment patterns for advanced or recurrent NSCLC in Japan reported a range of approaches [8]. Notably, no systemic treatment was administered in nearly 30% of patients overall and 50% of elderly patients. Based on the guideline recommendations [7], platinum-based combinations were the most common first-line therapy, and the use of non-platinum agents increased in the second-line setting, although platinum-based therapy continued to be used in about one-third of patients [8].

S-1 is an anticancer agent that combines tegafur (a pro-drug of 5-fluorouracil) with the modulators gimeracil and oteracil potassium. Gimeracil reversibly inhibits the 5-fluorouracil catabolic enzyme dihydropyrimidine dehydrogenase to help maintain effective 5-fluorouracil tissue concentrations, thus facilitating tumor-selective cytotoxicity. Oteracil potassium is distributed in high concentrations in gastrointestinal tissues and inhibits the activity of 5-fluorouracil at this site, thereby decreasing gastrointestinal toxicity [9, 10]. In a phase III study of Japanese chemotherapy-naïve patients with advanced NSCLC, S-1 + carboplatin was non-inferior to paclitaxel + carboplatin and was better tolerated [11]. In a subsequent phase III study in chemotherapy-naïve patients with advanced NSCLC, S-1 + cisplatin was non-inferior and better tolerated compared with docetaxel + cisplatin [12]. Thus, the combination of S-1 and platinum became an option for the first-line treatment of patients with advanced NSCLC. Other studies have highlighted the therapeutic effectiveness of S-1 monotherapy in previously treated NSCLC patients [13, 14].

The East Asia S-1 Trial in Lung Cancer (EAST-LC) was a randomized, non-inferiority, open-label, phase III study conducted at 84 medical centers in China, Japan, Hong Kong, Singapore, and Taiwan [15]. The trial demonstrated the non-inferiority of S-1 to standard docetaxel therapy in terms of overall survival (OS) in patients with advanced, previously treated NSCLC. A docetaxel dose-escalation study conducted in Japan found that the maximum tolerated dose of docetaxel was 70 mg/m^2^ and the recommended dose was 60 mg/m^2^ based on the occurrence of myelosuppression [16]. Based on the results of this study, Kunitoh et al. conducted a phase II study to evaluate the efficacy and toxicity of the initial treatment of NSCLC patients with docetaxel 60 mg/m^2^; the efficacy and safety of this dosage was confirmed for Japanese patients [17]. Subsequently, Mukohara et al. reported that the efficacy of docetaxel 60 mg/m^2^ in previously treated Japanese NSCLC patients was comparable to that obtained overseas with the conventional dose [18]. Additionally, the Japanese clinical practice guidelines recommend a dose of 60 mg/m^2^ as well [7]. Thus, docetaxel 60 mg/m^2^ was established as the standard dose for previously treated NSCLC in Japan, and Japanese medical centers in the EAST-LC study used the standard dose of docetaxel 60 mg/m^2^ [15]. The aim of our analysis was to assess the efficacy and safety of S-1 versus docetaxel 60 mg/m^2^ in the Japanese subgroup from the EAST-LC study.

## Patients and methods

### Study design and patients

The study design and patient eligibility criteria of the EAST-LC study were previously published [15]. Briefly, patients were aged ≥ 20 years; had locally advanced or metastatic NSCLC (clinical stage IIIB/IV, according to tumor-node-metastasis classification ver.7) with measurable or non-measurable lesions; had Eastern Cooperative Oncology Group performance status ≤ 2; and had received one or two previous chemotherapy regimens, including a platinum-based regimen or three previous regimens if patients had previously received gefitinib or erlotinib.

The study protocol was approved by the institutional review board/independent ethics committee at each study center and was conducted in accordance with International Conference on Harmonization Good Clinical Practice Guidelines, the ethical principles outlined in the Declaration of Helsinki, and applicable regulatory requirements in each country/region. All patients provided written informed consent prior to enrollment in the study. The study is registered with the Japan Pharmaceutical Information Center under the identification number, JapicCTI-101155.

The primary endpoint was OS, defined as the time from randomization to death from any cause. Secondary endpoints included progression-free survival (PFS), response rate (RR), quality of life (QOL), and incidence and severity of adverse events (AEs).

### Randomization and treatment

Randomization and treatment procedures were described in detail previously [15]. Patients were randomly assigned by a 1:1 ratio to receive S-1 or docetaxel using a web randomization system. The imbalance on the following factors was minimized: performance status (0–1/2); number of previous chemotherapy regimens (1/2/3); EGFR-tyrosine kinase inhibitor in previous treatments (yes/no); EGFR mutation status (yes/no/unknown); sex (male/female); histological type (squamous cell [SQ]/non-SQ carcinoma); smoking status [never/ever smoker (one or more cigarette smoked in a lifetime)]; and institution.

S-1 was given orally, twice daily, after meals, for 4 weeks in a 6-week schedule. The initial dose for patients receiving S-1 was 80 mg/day, 100 mg/day, or 120 mg/day, and was determined based on body surface area. Dose reductions were mandated in the S-1 group if the absolute neutrophil count was < 500/mm^3^, the platelet count was < 50,000/mm^3^, or grade 3 non-hematologic toxicities were present. At each time point, a 20-mg dose reduction was implemented if the patient met any of the abovementioned criteria, and the minimum dose was set as 80 mg/day. If patients required further dose reductions (less than 80 mg/day), they were withdrawn from the study. The docetaxel dose in Japanese patients was 60 mg/m^2^ and that in non-Japanese patients was 75 mg/m^2^, given intravenously on day 1 of a 3-week cycle. The docetaxel dose was reduced to 50 mg/m^2^ in Japanese patients and 60 mg/m^2^ in non-Japanese patients if any of the following were present: febrile neutropenia, platelet count < 25,000/mm^3^, grade 2 peripheral motor/sensory neuropathy or grade 3 non-hematologic toxicities. Patients who required a second dose reduction were withdrawn from the study.

### Assessments

Tumor imaging was performed every 6 weeks until radiological progression was confirmed. Imaging consisted of a computed tomographic scan, magnetic resonance imaging, or radiograph of the chest, abdomen, and head. Performance status, hematology, and biochemistry were assessed at baseline, on days 1 and 8 of cycle 1, on day 1 of each cycle thereafter, at end of therapy or patient withdrawal, and at 30 days after treatment discontinuation. QOL assessments were performed every 6 weeks and at end of therapy or patient withdrawal, using the European Organization for Research and Treatment of Cancer (EORTC) Quality of Life Questionnaire Core-30 (QLQ-C30).

### Statistical analysis

The full details of the overall statistical analysis have been reported previously [15]. However, in this study, statistical analyses were performed using SAS version 9.4 (SAS Institute Inc., Cary, NC, USA). Briefly, the OS and PFS rates were calculated using the Kaplan–Meier method; hazard ratios (HRs) were calculated by Cox proportional hazard model, including treatment as a covariate. RR values and associated two-sided 95% confidence intervals (CIs) were calculated. QOL variables were summarized descriptively with mean and standard error, and a linear mixed effect model was used to analyze changes over time.

The efficacy analysis and QOL assessments were based on the full analysis set, consisting of all randomized Japanese patients except those with a major protocol deviation. The safety analysis set consisted of patients who received at least one dose of the study drug.

## Results

### Patients

**Fig. 1:**
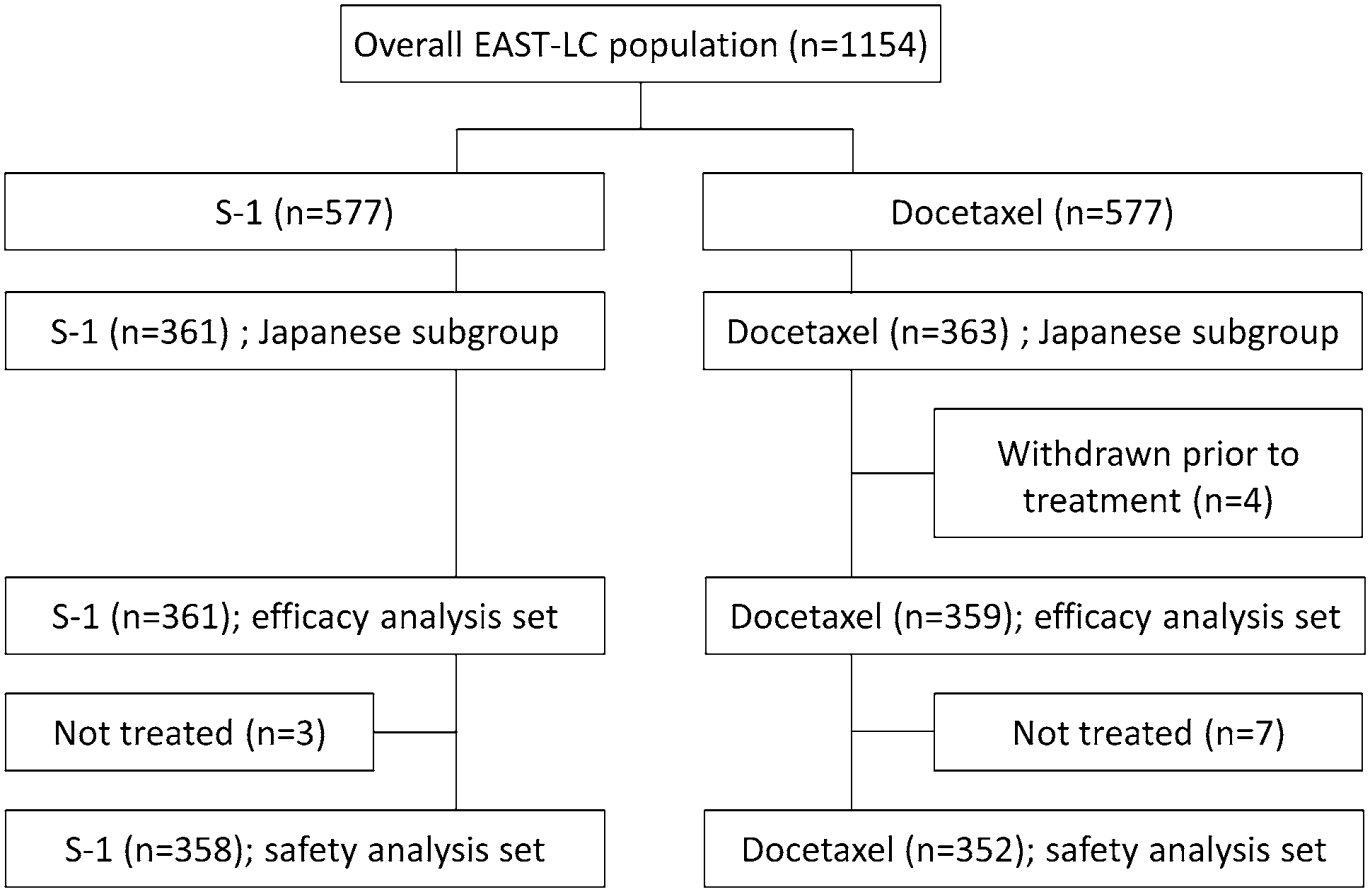
CONSORT diagram for patients enrolled at medical centers in Japan

A total of 724 patients were enrolled in Japanese medical centers (Supplementary material 1) participating in the EAST-LC study (361 received S-1 and 363 received docetaxel). The S-1 analysis set included all 361 randomized patients, and the safety analysis set included 358 patients (three were not treated). Four patients randomized to docetaxel were withdrawn prior to treatment and an additional seven were not treated. Thus, 359 and 352 patients, respectively, were included in the efficacy and safety analysis sets (Fig. 1).

The baseline characteristics of the Japanese patients are shown in Table 1. Characteristics of the Japanese patients enrolled in the EAST-LC study were well matched with those of the overall EAST-LC population [15]. The median relative dose intensity was slightly lower for S-1 versus docetaxel (89.0% vs 94.6%, respectively) (Table S1), similar to the overall EAST-LC population (92.2% vs 95.8%, respectively). Disease progression was the most common reason for treatment discontinuation in both groups (76.3% and 61.6% in S-1 and docetaxel groups, respectively), followed by AEs (9.8% and 19.0%, respectively) (Table S1). The rates for the other reasons of treatment discontinuation were similar between the Japanese subgroup and the overall EAST-LC population.

**Table 1:** Baseline characteristics of patients in the Japanese subgroup

	S-1 (*n* = 361)	Docetaxel (*n* = 359)
Age, years (range)	65 (23–85)	64 (36–82)
Histological type, *n* (%)		
Adenocarcinoma	263 (72.9)	270 (75.2)
Squamous cell carcinoma	67 (18.6)	65 (18.1)
Large cell carcinoma	10 (2.8)	3 (0.8)
Others	21 (5.8)	21 (5.8)
Number of previous treatments, *n* (%)		
1	233 (64.5)	235 (65.5)
2	101 (28.0)	101 (28.1)
3	27 (7.5)	23 (6.4)
EGFR status, *n* (%)		
Wild type	222 (61.5)	221 (61.6)
Mutant	75 (20.8)	70 (19.5)
Unknown	64 (17.7)	68 (18.9)
EGFR-TKI treatment history, *n* (%)		
No	281 (77.8)	286 (79.7)
Yes	80 (22.2)	73 (20.3)

Values are median (range) or number of patients (%)

*EGFR* endothelial growth factor receptor, *TKI* tyrosine kinase inhibitor

### Efficacy

**Fig. 2:**
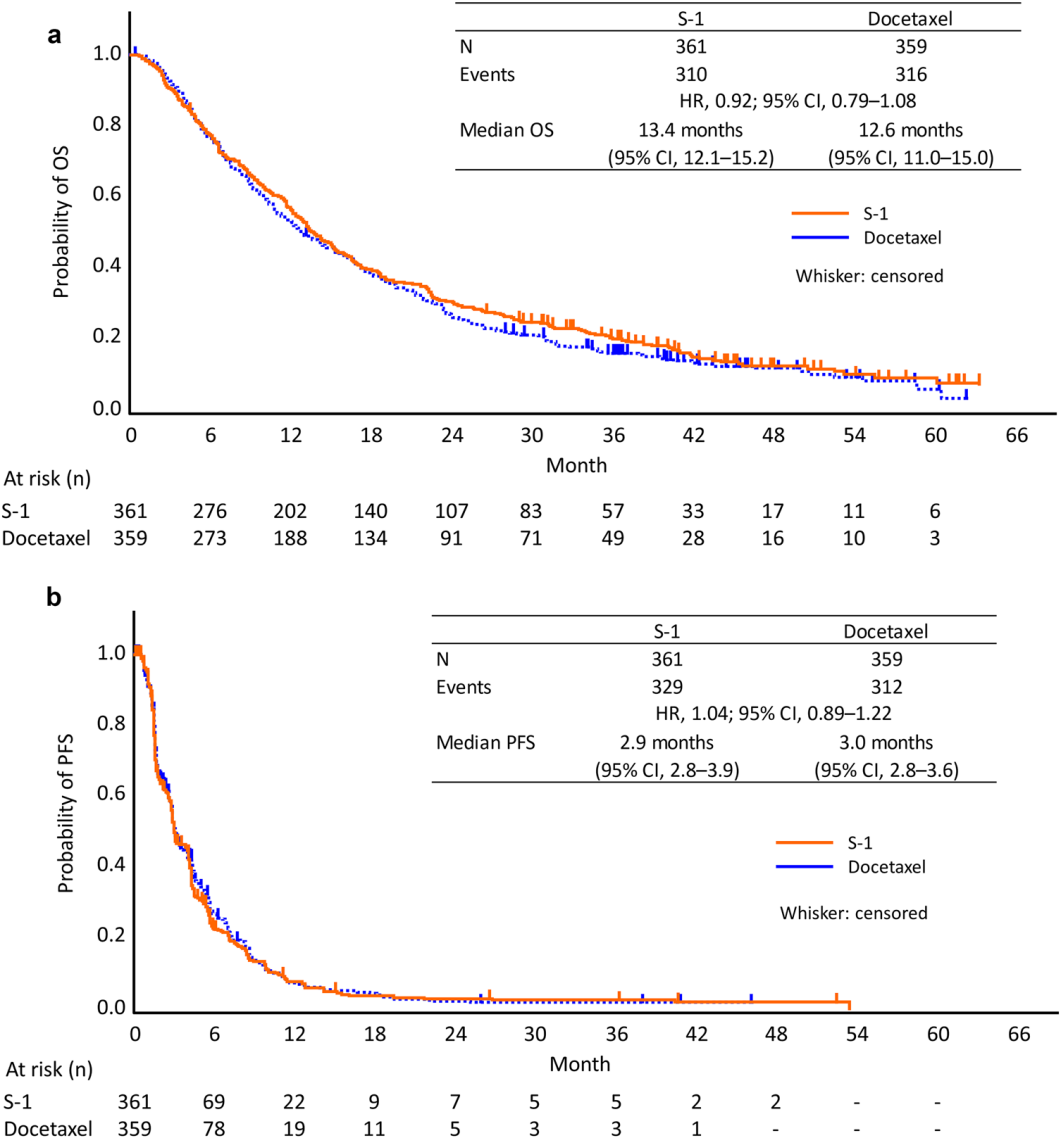
Kaplan–Meier curves for overall (**a**) and progression-free (**b**) survival. *HR* hazard ratio, *CI* confidence interval, *OS* overall survival, *PFS* progression-free survival

Median OS was 13.4 months in the S-1 group and 12.6 months in the docetaxel group (Fig. 2a). The HR was 0.92 (95% CI: 0.79–1.08) and the upper limit of the HR fell below the non-inferiority margin of 1.2 as well as in the overall EAST-LC population [15].

Kaplan–Meier curves for PFS are shown in Fig. 2b. Median PFS was almost identical in the S-1 and docetaxel groups (2.9 and 3.0 months, respectively; HR 1.04; 95% CI: 0.89–1.22), which was again consistent with the results for the overall EAST-LC population.

The RR was 9.4% and 10.3% in S-1 and docetaxel groups, respectively. The RR was similar in both groups, and it was also similar to the results of the overall EAST-LC population.

**Fig. 3:**
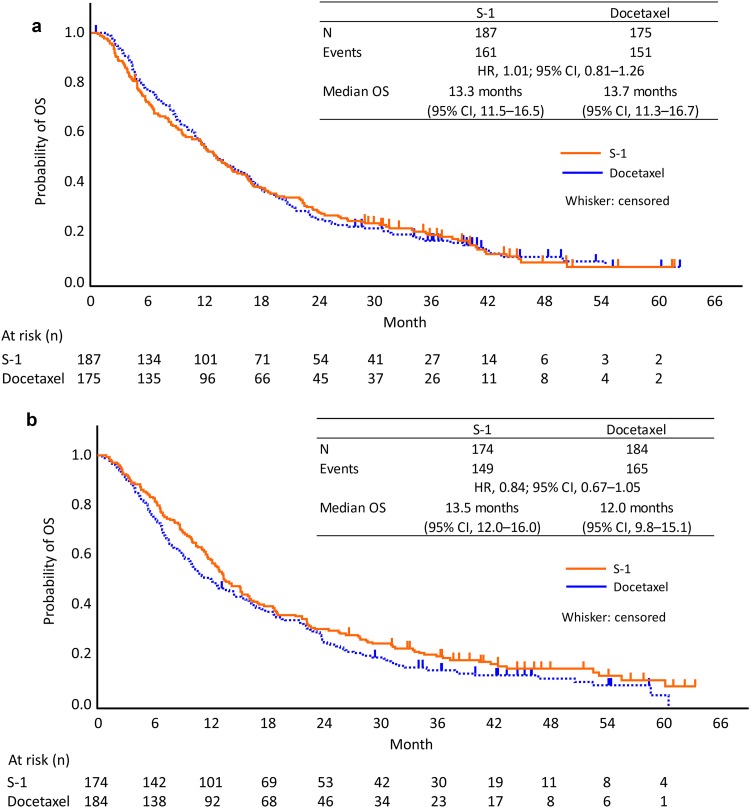
Kaplan–Meier curves for overall survival (OS) in patients with (**a**) or without (**b**) pemetrexed pretreatment. *HR* hazard ratio, *CI* confidence interval

Forest plots for OS outcomes by each factor are shown in Figure S1. There were no differences in OS between the S-1 and docetaxel groups in any of the patient subgroups analyzed, similar to the overall EAST-LC population results. In patients pretreated with pemetrexed (HR 1.01; 95% CI: 0.81–1.26) (Fig. 3a) or without pemetrexed (HR 0.84; 95% CI: 0.67–1.05) (Fig. 3b), S-1 and docetaxel had equivalent efficacy in terms of OS. The results also showed that treatment with taxanes had no influence on the efficacy of docetaxel (Figure S1).

**Fig. 4:**
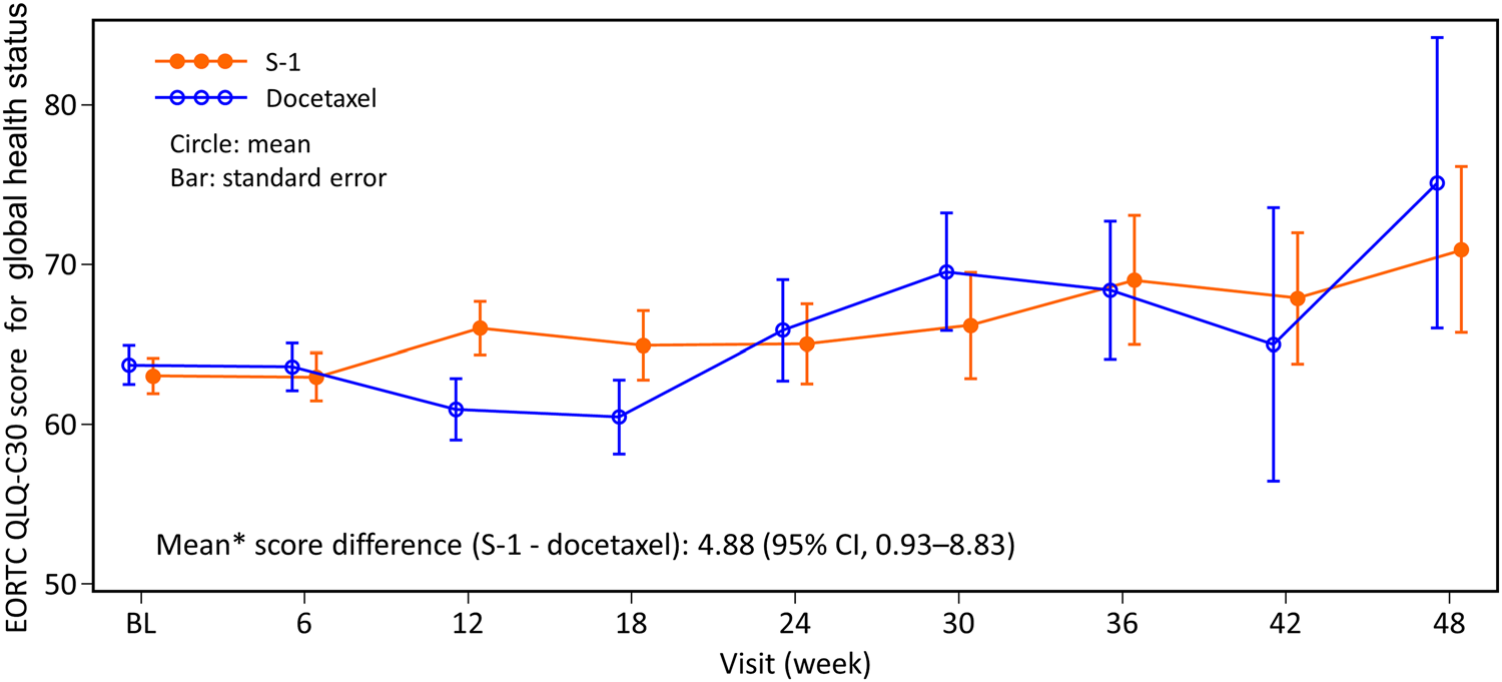
Change in EORTC QLQ-C30 score for global health status. *Adjusted mean based on the model statistics. EORTC QLQ-C30, European Organization for Research and Treatment of Cancer Quality of Life Questionnaire Core-30; *QOL* quality of life *CI* confidence interval, *BL* baseline

EORTC QLQ-C30 global health status was favored in the S-1 group. The adjusted mean score difference (S-1 − docetaxel) based on the linear model was 4.88 (95% CI: 0.93–8.83) (Fig. 4), which is comparable to that of the overall population [15].

### Safety and AEs

A summary of AEs occurring in Japanese patients treated with S-1 or docetaxel is provided in Table 2. Regarding the most common AEs of any grade in each group, AE profiles in the Japanese subgroup and the overall EAST-LC population were similar. The proportion of patients with grade ≥ 3 febrile neutropenia was markedly lower in the S-1 versus docetaxel group (1.1% vs 15.1%); a similar trend was observed for grade ≥ 3 leucopenia (1.7% vs 32.1%) and neutropenia (6.7% vs 54.0%). In contrast, grade ≥ 3 decreased appetite and diarrhea were more common in the S-1 group compared with the docetaxel group (9.8% vs 3.4% and 8.4% vs 0.9%, respectively). Rates of other hematologic and non-hematologic AEs were similar in the S-1 and docetaxel groups. No grade 5 hematologic toxicities occurred. One treatment-related death occurred in the S-1 group in the Japanese subgroup (hypovolemic shock) [15].

**Table 2:** Summary of adverse events, including hematologic and non-hematologic toxicities

Number of patients (%)	S-1 (*n* = 358)		Docetaxel (*n* = 352)	
	All grades	Grade ≥ 3	All grades	Grade ≥ 3
Hematologic toxicities				
Leucopenia	23 (6.4)	6 (1.7)	145 (41.2)	113 (32.1)
Neutropenia	50 (14.0)	24 (6.7)	200 (56.8)	190 (54.0)
Febrile neutropenia	4 (1.1)	4 (1.1)	53 (15.1)	53 (15.1)
Anemia	35 (9.8)	10 (2.8)	22 (6.3)	4 (1.1)
Thrombocytopenia	38 (10.6)	6 (1.7)	9 (2.6)	0 (0.0)
Non-hematologic toxicities				
Stomatitis	112 (31.3)	12 (3.4)	68 (19.3)	4 (1.1)
Nausea	147 (41.1)	5 (1.4)	110 (31.3)	6 (1.7)
Vomiting	61 (17.0)	7 (2.0)	44 (12.5)	3 (0.9)
Decreased appetite	211 (58.9)	35 (9.8)	163 (46.3)	12 (3.4)
Diarrhea	164 (45.8)	30 (8.4)	73 (20.7)	3 (0.9)
Constipation	70 (19.6)	3 (0.8)	86 (24.4)	1 (0.3)
Maculopapular rash	57 (15.9)	5 (1.4)	46 (13.1)	1 (0.3)
Skin hyperpigmentation	123 (34.4)	–	9 (2.6)	–
Peripheral edema	11 (3.0)	0 (0.0)	75 (21.3)	3 (0.9)
Pyrexia	54 (15.0)	0 (0.0)	59 (16.8)	0 (0.0)
Weight loss	50 (14.0)	2 (0.6)	18 (5.1)	0 (0.0)
Peripheral sensory neuropathy	25 (7.0)	1 (0.3)	69 (19.6)	3 (0.9)
Cough	21 (5.9)	0 (0.0)	28 (8.0)	0 (0.0)
Dyspnea	14 (3.9)	2 (0.6)	22 (6.3)	2 (0.6)
Alopecia	8 (2.2)	–	205 (58.2)	–

## Discussion

The efficacy and safety results of this Japanese subgroup analysis are almost equivalent to those in the overall EAST-LC population [15]. Because myelosuppression by docetaxel is expressed more strongly in Japanese compared with Western patients, docetaxel 60 mg/m^2^ was established as the standard dose in Japan [16–18]. Subsequently, several phase III studies in previously treated Japanese NSCLC patients used the arm receiving docetaxel 60 mg/m^2^ as the control arm [19–21]. In those studies, docetaxel efficacy was similar to the efficacy observed in overseas phase III studies with the conventional dose [22, 23]; thus, docetaxel 60 mg/m^2^ was confirmed as the standard docetaxel dose in Japan. In the present subgroup analysis, S-1 and the standard dose (60 mg/m^2^) of docetaxel in Japan showed equivalent efficacy; thus, treatment with S-1 for previously treated patients with NSCLC is expected to translate into important clinical benefits.

The safety profile of S-1 and docetaxel in this Japanese subgroup analysis was also consistent with that in the overall EAST-LC population [15]. Hematologic toxicities were much more common with docetaxel, whereas a higher proportion of S-1 recipients experienced gastrointestinal toxicities. The hematologic toxicity profile of docetaxel in the overall EAST-LC population and the Japanese subgroup was similar to that reported in other large, randomized clinical trials of docetaxel in previously treated patients with advanced NSCLC [19–21].

Regarding the differences in QOL between the S-1 and docetaxel groups, the numerical difference in EORTC QLQ-C30 score for global health status between the groups was 4.88, which is higher than the value of 4 that has been reported as the small difference [24]. This suggests that the QOL of patients treated with S-1 might be clinically meaningful compared with that of patients treated with docetaxel.

The current results also confirmed that the efficacy of S-1 in patients pretreated with pemetrexed (inhibitor of thymidylate synthase widely used in patients with NSCLC) was similar to that of docetaxel. Although some retrospective investigations showed that previous treatment with pemetrexed had no influence on the efficacy of S-1 [25, 26], this is the first prospective clinical trial data indicating that S-1 is effective in patients pretreated with combination chemotherapy regimens including pemetrexed. Thus, S-1 is a suitable option for NSCLC patients in current clinical practice who received combination chemotherapy regimens including pemetrexed as initial treatment.

The results of this study need to be interpreted in light of several limitations. First, the current report is a subgroup analysis of a larger data set, resulting in a potential loss of statistical power associated with smaller patient numbers and multiple comparisons. In addition, the EAST-LC study was conducted at a time when newer agents, such as ramucirumab and immune checkpoint inhibitors, were not routinely available. Docetaxel was chosen as the comparator for S-1 based on clinical practice at the time the study was designed; however, second-line therapy treatment options now differ given the greater effectiveness of newer agents and combinations compared with docetaxel [27–30]. Nevertheless, targeted therapies and immunotherapies are only appropriate for specific subgroups of patients, and these agents are not universally effective. Therefore, there remains a clinical need for effective chemotherapy options such as S-1.

In conclusion, this Japanese subgroup analysis of the EAST-LC study showed similar efficacy with S-1 and docetaxel. S-1 was associated with less hematologic toxicity, especially febrile neutropenia, than docetaxel, whereas gastrointestinal toxicity was more common with S-1 than docetaxel. The results also showed similar prolonged OS with S-1 and docetaxel, despite pretreatment with pemetrexed. Additionally, regarding QOL, S-1 showed clinically significant results. Based on the results of this study, S-1 may be a suitable option as a second-line or later-line treatment for advanced NSCLC patients in Japan.

## Electronic supplementary material

Below is the link to the electronic supplementary material.

Supplementary material 1 (DOCX 14 KB)

Supplementary material 2 (DOCX 160 KB)

Supplementary material 3 (DOCX 15 KB)
