# Supplementary material for: Japanese subgroup analysis of a phase III study of S-1 versus docetaxel in non-small cell lung cancer patients after platinum-based treatment: EAST-LC
Source: Int J Clin Oncol. 2019 Mar 4;24(5):485–93. doi: 10.1007/s10147-019-01396-z (PMC6469651; doi:10.1007/s10147-019-01396-z)
Supplement: Supplementary file 1 — Supplementary material 1 (DOCX 14 KB) [file 10147_2019_1396_MOESM1_ESM.docx]

**Supplemental material 1. Collaborating medical centers in Japan**

Hokkaido University Hospital, Sendai Kousei Hospital, Miyagi Cancer Center, Tohoku University Hospital, Fukushima Medical University Hospital, Saitama Cancer Center, Niigata University Medical & Dental Hospital, Niigata Cancer Center Hospital, National Cancer Center Hospital East, Chiba University Hospital, Chiba Cancer Center, Ibaraki Prefectural Center Hospital, National Cancer Center Hospital, Nippon Medical School Hospital, Tokyo Medical University Hospital, Tokyo Metropolitan Cancer and Infectious Diseases Center Komagome Hospital, The Cancer Institute Hospital of JFCR, Toranomon Hospital, Fukujuji Hospital, Yokohama Municipal Citizen’s Hospital, Kanagawa Cardiovascular and Respiratory Center, Shizuoka Cancer Center, Nagoya Medical Center, Aichi Cancer Center Hospital, Aichi Cancer Center Aichi Hospital, Kanazawa University Hospital, Kouseiren Takaoka Hospital, Kindai University Hospital, Osaka City General Hospital, Osaka International Cancer Institute, Osaka Habikino Medical Center, Osaka City University Hospital, Toneyama National Hospital, Kobe City Medical Center General Hospital, Hyogo Cancer Center, Okayama University Hospital, Kurashiki Central Hospital, Hiroshima City Hiroshima Citizens Hospital, Yamaguchi-Ube Medical Center, Shikoku Cancer Center, Kyushu Cancer Center, Kyushu University Hospital, Aso Iizuka Hospital, Nagasaki University Hospital, Japanese Red Cross Nagasaki Genbaku Hospital, Nagasaki Medical Center, and Kumamoto Regional Medical Center.
