# Supplementary material for: Japanese subgroup analysis of a phase III study of S-1 versus docetaxel in non-small cell lung cancer patients after platinum-based treatment: EAST-LC
Source: Int J Clin Oncol. 2019 Mar 4;24(5):485–93. doi: 10.1007/s10147-019-01396-z (PMC6469651; doi:10.1007/s10147-019-01396-z)
Supplement: Supplementary file 2 — Supplementary material 2 (DOCX 160 KB) [file 10147_2019_1396_MOESM2_ESM.docx]

**Figure S1.** Forest plot for overall survival (OS) in the Japanese subgroup.


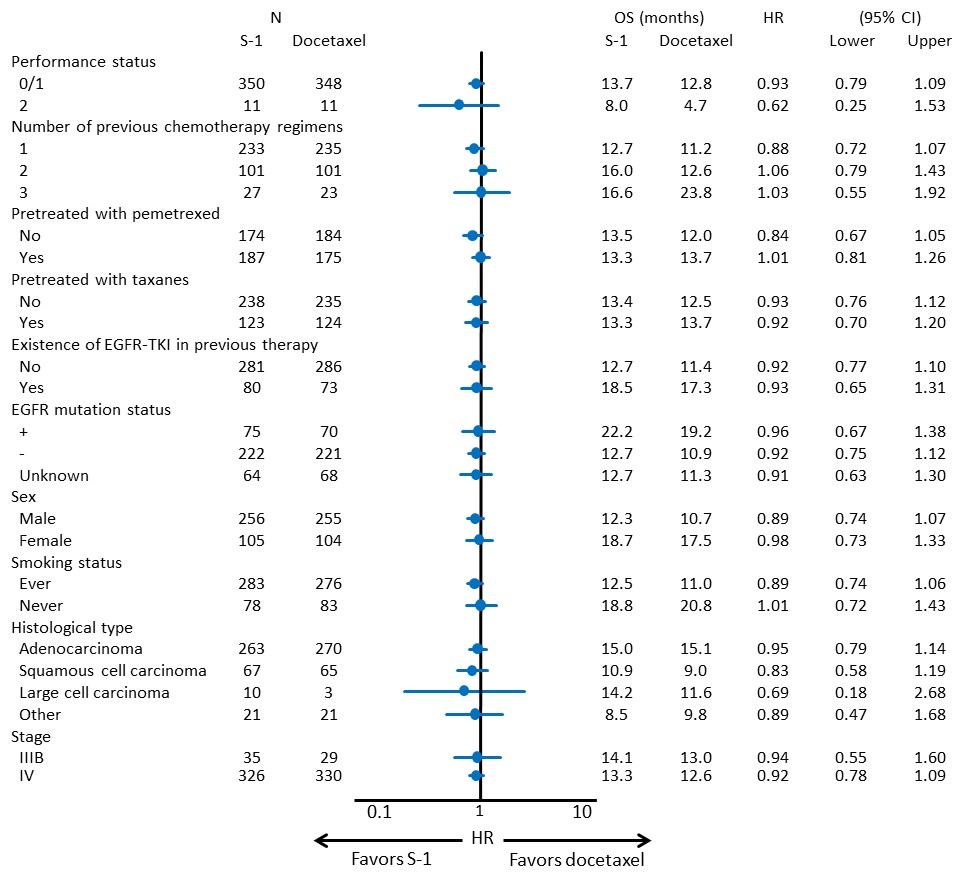


Abbreviations: HR, hazard ratio; CI, confidence interval; EGFR, endothelial growth factor receptor; TKI, tyrosine kinase inhibitor.
