# Supplementary material for: Japanese subgroup analysis of a phase III study of S-1 versus docetaxel in non-small cell lung cancer patients after platinum-based treatment: EAST-LC
Source: Int J Clin Oncol. 2019 Mar 4;24(5):485–93. doi: 10.1007/s10147-019-01396-z (PMC6469651; doi:10.1007/s10147-019-01396-z)
Supplement: Supplementary file 3 — Supplementary material 3 (DOCX 15 KB) [file 10147_2019_1396_MOESM3_ESM.docx]

**Table S1.** Treatment duration, dose intensity, and discontinuations

|  | **S-1 (n=358)** | **Docetaxel (n=352)** |
| --- | --- | --- |
| Number of cycles, median (range) | 2 (1–19) | 3 (1–41) |
| Median relative dose intensity, % | 89.0 | 94.6 |
| Reasons for treatment discontinuation, n (%) |  |  |
| Progressive disease | 273 (76.3) | 217 (61.6) |
| Adverse events^1^ | 35 (9.8) | 67 (19.0) |
| Refused treatment | 21 (5.9) | 24 (6.8) |
| Others^2^ | 29 (8.1) | 44 (12.5) |

^1^ Grade ≥3 peripheral nerve disease, grade ≥2 pneumonitis, or grade ≥4 non-hematologic events, based on investigator’s assessment.

^2^ Including patients who could not start the next course of treatment within 14 days of the scheduled start date, those who required a dosage decrease greater than that allowed in the protocol, patients who changed residence or transferred to other hospitals, patients no longer eligible for treatment, or physician’s decision.
